# 2-(Naphthalen-2-yl­oxy)-5-nitro­pyridine

**DOI:** 10.1107/S1600536811044060

**Published:** 2011-10-29

**Authors:** Shah Bakhtiar Nasir, Zainal Abidin Fairuz, Zanariah Abdullah, Seik Weng Ng, Edward R. T. Tiekink

**Affiliations:** a Department of Chemistry, University of Malaya, 50603 Kuala Lumpur, Malaysia; b Chemistry Department, Faculty of, Science, King Abdulaziz University, PO Box 80203 Jeddah, Saudi Arabia

## Abstract

A nearly orthogonal relationship is found for the ring systems in the title compound, C_15_H_10_N_2_O_3_, with the dihedral angle between the rings being 86.13 (11)°. The nitro group is approximately coplanar with the pyridine ring to which it is connected [the O—N—C—C torsion angle = −1.8 (4)°]. This coplanarity allows for the close approach of these residues in the crystal structure enabling the formation of N—O⋯π(pyridine) inter­actions [3.547 (4) Å]. Further consolidation of the crystal packing is afforded by weak π–π inter­actions [centroid–centroid distances = 3.9576 (16) and 3.9822 (16) Å].

## Related literature

For the structure of a related nitro­pyridine derivative, see: Nasir *et al.* (2010 ▶). For discussion on nitro-O⋯π inter­actions, see: Huang *et al.* (2008 ▶).
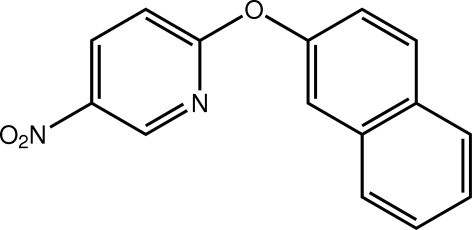


## Experimental

### 

#### Crystal data

C_15_H_10_N_2_O_3_
*M* *_r_* = 266.25
Monoclinic, 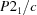

*a* = 6.7389 (12) Å
*b* = 8.9182 (16) Å
*c* = 21.072 (4) Å
β = 94.289 (3)°
*V* = 1262.9 (4) Å^3^
*Z* = 4
Mo *K*α radiation
μ = 0.10 mm^−1^
*T* = 293 K
0.25 × 0.20 × 0.15 mm

#### Data collection

Bruker SMART APEX diffractometer
Absorption correction: multi-scan (*SADABS*; Sheldrick, 1996 ▶) *T*_min_ = 0.976, *T*_max_ = 0.985
6512 measured reflections
2224 independent reflections
1052 reflections with *I* > 2σ(*I*)
*R*_int_ = 0.052

#### Refinement

*R*[*F* ^2^ > 2σ(*F* ^2^)] = 0.054
*wR*(*F* ^2^) = 0.193
*S* = 0.94
2224 reflections
158 parameters
H-atom parameters constrained
Δρ_max_ = 0.26 e Å^−3^
Δρ_min_ = −0.18 e Å^−3^

### 

Data collection: *APEX2* (Bruker, 2009 ▶); cell refinement: *SAINT* (Bruker, 2009 ▶); data reduction: *SAINT*; program(s) used to solve structure: *SHELXS97* (Sheldrick, 2008 ▶); program(s) used to refine structure: *SHELXL97* (Sheldrick, 2008 ▶); molecular graphics: *ORTEP-3* (Farrugia, 1997 ▶) and *DIAMOND* (Brandenburg, 2006 ▶); software used to prepare material for publication: *publCIF* (Westrip, 2010 ▶).

## Supplementary Material

Crystal structure: contains datablock(s) global, I. DOI: 10.1107/S1600536811044060/hg5118sup1.cif

Structure factors: contains datablock(s) I. DOI: 10.1107/S1600536811044060/hg5118Isup2.hkl

Supplementary material file. DOI: 10.1107/S1600536811044060/hg5118Isup3.cml

Additional supplementary materials:  crystallographic information; 3D view; checkCIF report

## Figures and Tables

**Fig. 1.**
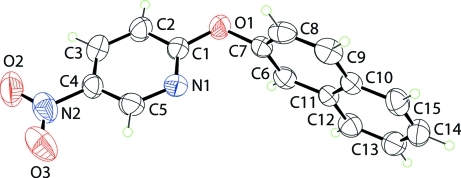
The molecular structure of (I) showing the atom-labelling scheme and displacement ellipsoids at the 35% probability level.

**Fig. 2.**
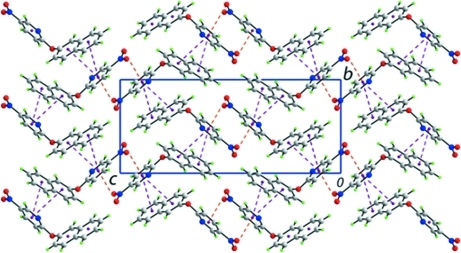
Unit-cell contents for (I) shown in projection down the a axis. The N—O···π and π–π interactions are shown as orange and purple dashed lines, respectively.

## supplementary crystallographic information

### Comment

In continuation of structural studies of nitro-pyridine derivatives (Nasir *et al.*, 2010), the title compound was synthesized and characterized crystallographically. The molecule of (I), Fig. 1, is highly twisted as seen in the near orthogonal relationship between the two ring systems with the dihedral angle formed between the nitro-pyridine ring and naphthalyl ring being 86.13 (11)°. The nitro group is co-planar with the pyridyl ring to which it is attached with the O2—N2—C4—C3 torsion angle being -1.8 (4)°.

The most prominent feature of the crystal packing appears to be N—O···π interactions (Huang *et al.*, 2008). The distance between O3···π(pyridyl) = 3.547 (4) Å, with the N2—O3···ring centroid(pyridyl)^i^ angle = 81.9 (2)° for symmetry operation *i*: 1 - *x*, 1 - *y*, 1 - *z*. Similar interactions were reported in the crystal structure of 2-(4-methoxyphenoxy)-3-nitropyridine (Nasir *et al.*, 2010). In (I), the N—O···π interactions along with weak π–π interactions, whereby both rings of the naphthalyl residue interact with the pyridyl ring, consolidate the crystal packing. The parameters associated with the π–π interactions are ring centroid(pyridyl)···ring centroid(C6–C11) and (C10–C15) = 3.9822 (16) and 3.9576 (16) Å, respectively, for *ii*: 1 - *x*, -1/2 + *y*, 3/2 - *z*. A view of the crystal packing is shown in Fig. 2.

### Experimental

2-Naphthol (2.88 g, 20 mmol) and sodium hydroxide (0.80 g, 20 mmol) were dissolved in water (50 ml) and to the solution was added 2-chloro-5-nitropyridine (3.17 g, 20 mmol) dissolved in THF (50 ml). The mixture was heated for 7 h. Water was added and the organic phase extracted with chloroform. The chloroform solution was dried over sodium sulfate; slow evaporation led to the formation of colourless crystals.

### Refinement

Hydrogen atoms were placed at calculated positions (C—H 0.93 Å) and were treated as riding on their parent carbon atoms, with *U*(H) set to 1.2*U*_eq_(C). The naphthalene fused-ring was refined as two fused hexagons with C≐C distances fixed at 1.39 Å.

### Crystal data

**Table:**

C_15_H_10_N_2_O_3_	*F*(000) = 552
*M**_r_* = 266.25	*D*_x_ = 1.400 Mg m^−^^3^
Monoclinic, *P*2_1_/*c*	Mo *K*α radiation, λ = 0.71073 Å
Hall symbol: -P 2ybc	Cell parameters from 1499 reflections
*a* = 6.7389 (12) Å	θ = 2.5–21.3°
*b* = 8.9182 (16) Å	µ = 0.10 mm^−^^1^
*c* = 21.072 (4) Å	*T* = 293 K
β = 94.289 (3)°	Block, colourless
*V* = 1262.9 (4) Å^3^	0.25 × 0.20 × 0.15 mm
*Z* = 4	

### Data collection

**Table:**

Bruker SMART APEX diffractometer	2224 independent reflections
Radiation source: fine-focus sealed tube	1052 reflections with *I* > 2σ(*I*)
graphite	*R*_int_ = 0.052
ω scans	θ_max_ = 25.0°, θ_min_ = 1.9°
Absorption correction: multi-scan (*SADABS*; Sheldrick, 1996)	*h* = −8→7
*T*_min_ = 0.976, *T*_max_ = 0.985	*k* = −9→10
6512 measured reflections	*l* = −23→25

### Refinement

**Table:**

Refinement on *F*^2^	Secondary atom site location: difference Fourier map
Least-squares matrix: full	Hydrogen site location: inferred from neighbouring sites
*R*[*F*^2^ > 2σ(*F*^2^)] = 0.054	H-atom parameters constrained
*wR*(*F*^2^) = 0.193	*w* = 1/[σ^2^(*F*_o_^2^) + (0.1*P*)^2^] where *P* = (*F*_o_^2^ + 2*F*_c_^2^)/3
*S* = 0.94	(Δ/σ)_max_ = 0.001
2224 reflections	Δρ_max_ = 0.26 e Å^−^^3^
158 parameters	Δρ_min_ = −0.18 e Å^−^^3^
0 restraints	Extinction correction: *SHELXL97* (Sheldrick, 2008), Fc^*^=kFc[1+0.001xFc^2^λ^3^/sin(2θ)]^-1/4^
Primary atom site location: structure-invariant direct methods	Extinction coefficient: 0.024 (5)

### Fractional atomic coordinates and isotropic or equivalent isotropic displacement parameters (Å^2^)

**Table:**

	*x*	*y*	*z*	*U*_iso_*/*U*_eq_	
O1	0.5816 (3)	0.6709 (3)	0.69203 (11)	0.0864 (8)	
O2	0.8327 (5)	0.2782 (3)	0.47200 (13)	0.1210 (11)	
O3	0.5363 (6)	0.1958 (4)	0.48260 (14)	0.1319 (12)	
N1	0.4532 (4)	0.4809 (3)	0.62772 (12)	0.0709 (8)	
N2	0.6776 (7)	0.2766 (4)	0.49811 (14)	0.0929 (10)	
C1	0.6029 (5)	0.5686 (4)	0.64546 (14)	0.0662 (8)	
C2	0.7856 (5)	0.5678 (4)	0.61964 (15)	0.0796 (10)	
H2	0.8868	0.6323	0.6347	0.096*	
C3	0.8131 (5)	0.4702 (4)	0.57165 (15)	0.0804 (10)	
H3	0.9339	0.4653	0.5531	0.097*	
C4	0.6578 (6)	0.3793 (3)	0.55143 (13)	0.0663 (9)	
C5	0.4845 (6)	0.3863 (3)	0.58025 (15)	0.0747 (10)	
H5	0.3819	0.3219	0.5662	0.090*	
C6	0.41265 (19)	0.58487 (18)	0.78172 (8)	0.0638 (8)	
H6	0.5213	0.5246	0.7941	0.077*	
C7	0.4128 (2)	0.6695 (2)	0.72633 (7)	0.0638 (8)	
C8	0.2505 (3)	0.75959 (18)	0.70776 (6)	0.0727 (9)	
H8	0.2506	0.8162	0.6707	0.087*	
C9	0.0879 (2)	0.76505 (16)	0.74459 (7)	0.0740 (9)	
H9	−0.0208	0.8253	0.7322	0.089*	
C10	0.08767 (17)	0.68042 (13)	0.79997 (6)	0.0565 (8)	
C11	0.25006 (17)	0.59033 (13)	0.81854 (6)	0.0521 (7)	
C12	0.2499 (3)	0.50569 (17)	0.87393 (7)	0.0714 (9)	
H12	0.3585	0.4454	0.8863	0.086*	
C13	0.0873 (3)	0.5111 (2)	0.91075 (7)	0.0822 (10)	
H13	0.0872	0.4545	0.9478	0.099*	
C14	−0.0751 (3)	0.6012 (2)	0.89219 (8)	0.0864 (11)	
H14	−0.1839	0.6049	0.9168	0.104*	
C15	−0.07492 (19)	0.6859 (2)	0.83680 (9)	0.0745 (10)	
H15	−0.1836	0.7461	0.8244	0.089*	

### Atomic displacement parameters (Å^2^)

**Table:**

	*U*^11^	*U*^22^	*U*^33^	*U*^12^	*U*^13^	*U*^23^
O1	0.0829 (16)	0.0914 (17)	0.0898 (16)	−0.0342 (13)	0.0385 (13)	−0.0301 (14)
O2	0.158 (3)	0.121 (2)	0.089 (2)	0.032 (2)	0.0437 (19)	−0.0121 (16)
O3	0.164 (3)	0.118 (2)	0.110 (2)	−0.007 (2)	−0.016 (2)	−0.0479 (19)
N1	0.0775 (18)	0.0660 (17)	0.0704 (17)	−0.0159 (14)	0.0136 (14)	−0.0087 (14)
N2	0.133 (3)	0.083 (2)	0.062 (2)	0.023 (2)	0.000 (2)	−0.0026 (18)
C1	0.074 (2)	0.067 (2)	0.0588 (19)	−0.0110 (17)	0.0132 (16)	−0.0046 (16)
C2	0.073 (2)	0.094 (2)	0.074 (2)	−0.0240 (19)	0.0173 (18)	−0.012 (2)
C3	0.085 (3)	0.095 (3)	0.063 (2)	0.000 (2)	0.0180 (19)	0.003 (2)
C4	0.089 (3)	0.063 (2)	0.0475 (18)	0.0060 (18)	0.0089 (17)	0.0015 (15)
C5	0.092 (3)	0.065 (2)	0.067 (2)	−0.0149 (18)	0.0039 (19)	−0.0038 (17)
C6	0.0594 (19)	0.0561 (18)	0.075 (2)	0.0043 (14)	0.0001 (16)	−0.0062 (16)
C7	0.068 (2)	0.0581 (19)	0.066 (2)	−0.0146 (15)	0.0123 (16)	−0.0168 (16)
C8	0.090 (2)	0.061 (2)	0.064 (2)	−0.0162 (18)	−0.0125 (18)	0.0053 (16)
C9	0.064 (2)	0.069 (2)	0.086 (2)	0.0077 (16)	−0.0115 (18)	0.0010 (18)
C10	0.0562 (18)	0.0519 (17)	0.0608 (18)	0.0013 (14)	0.0011 (15)	−0.0056 (14)
C11	0.0529 (17)	0.0497 (16)	0.0535 (17)	0.0008 (13)	0.0023 (13)	−0.0044 (14)
C12	0.083 (2)	0.065 (2)	0.066 (2)	0.0065 (16)	0.0013 (18)	0.0028 (16)
C13	0.114 (3)	0.073 (2)	0.061 (2)	−0.008 (2)	0.016 (2)	0.0011 (17)
C14	0.077 (3)	0.094 (3)	0.092 (3)	−0.009 (2)	0.026 (2)	−0.022 (2)
C15	0.062 (2)	0.077 (2)	0.085 (2)	0.0101 (16)	0.0050 (18)	−0.0151 (19)

### Geometric parameters (Å, °)

**Table:**

O1—C1	1.355 (3)	C6—H6	0.9300
O1—C7	1.393 (2)	C7—C8	1.3900
O2—N2	1.217 (4)	C8—C9	1.3900
O3—N2	1.219 (4)	C8—H8	0.9300
N1—C1	1.309 (4)	C9—C10	1.3900
N1—C5	1.337 (4)	C9—H9	0.9300
N2—C4	1.463 (4)	C10—C11	1.3900
C1—C2	1.383 (4)	C10—C15	1.3900
C2—C3	1.358 (4)	C11—C12	1.3900
C2—H2	0.9300	C12—C13	1.3900
C3—C4	1.366 (4)	C12—H12	0.9300
C3—H3	0.9300	C13—C14	1.3900
C4—C5	1.358 (4)	C13—H13	0.9300
C5—H5	0.9300	C14—C15	1.3900
C6—C7	1.3900	C14—H14	0.9300
C6—C11	1.3900	C15—H15	0.9300
			
C1—O1—C7	120.3 (2)	C8—C7—O1	120.35 (16)
C1—N1—C5	115.4 (3)	C7—C8—C9	120.0
O2—N2—O3	124.5 (4)	C7—C8—H8	120.0
O2—N2—C4	118.0 (4)	C9—C8—H8	120.0
O3—N2—C4	117.5 (4)	C10—C9—C8	120.0
N1—C1—O1	119.2 (3)	C10—C9—H9	120.0
N1—C1—C2	125.1 (3)	C8—C9—H9	120.0
O1—C1—C2	115.7 (3)	C9—C10—C11	120.0
C3—C2—C1	118.1 (3)	C9—C10—C15	120.0
C3—C2—H2	120.9	C11—C10—C15	120.0
C1—C2—H2	120.9	C12—C11—C10	120.0
C2—C3—C4	118.0 (3)	C12—C11—C6	120.0
C2—C3—H3	121.0	C10—C11—C6	120.0
C4—C3—H3	121.0	C13—C12—C11	120.0
C5—C4—C3	119.8 (3)	C13—C12—H12	120.0
C5—C4—N2	120.2 (4)	C11—C12—H12	120.0
C3—C4—N2	119.9 (3)	C12—C13—C14	120.0
N1—C5—C4	123.6 (3)	C12—C13—H13	120.0
N1—C5—H5	118.2	C14—C13—H13	120.0
C4—C5—H5	118.2	C15—C14—C13	120.0
C7—C6—C11	120.0	C15—C14—H14	120.0
C7—C6—H6	120.0	C13—C14—H14	120.0
C11—C6—H6	120.0	C14—C15—C10	120.0
C6—C7—C8	120.0	C14—C15—H15	120.0
C6—C7—O1	119.52 (16)	C10—C15—H15	120.0
			
C5—N1—C1—O1	178.1 (3)	C1—O1—C7—C8	−94.2 (3)
C5—N1—C1—C2	−1.0 (5)	C6—C7—C8—C9	0.0
C7—O1—C1—N1	8.4 (4)	O1—C7—C8—C9	−175.77 (15)
C7—O1—C1—C2	−172.4 (3)	C7—C8—C9—C10	0.0
N1—C1—C2—C3	0.8 (5)	C8—C9—C10—C11	0.0
O1—C1—C2—C3	−178.3 (3)	C8—C9—C10—C15	180.0
C1—C2—C3—C4	0.5 (5)	C9—C10—C11—C12	180.0
C2—C3—C4—C5	−1.4 (5)	C15—C10—C11—C12	0.0
C2—C3—C4—N2	177.5 (3)	C9—C10—C11—C6	0.0
O2—N2—C4—C5	177.1 (3)	C15—C10—C11—C6	180.0
O3—N2—C4—C5	−2.4 (5)	C7—C6—C11—C12	180.0
O2—N2—C4—C3	−1.8 (4)	C7—C6—C11—C10	0.0
O3—N2—C4—C3	178.7 (3)	C10—C11—C12—C13	0.0
C1—N1—C5—C4	−0.1 (5)	C6—C11—C12—C13	180.0
C3—C4—C5—N1	1.3 (5)	C11—C12—C13—C14	0.0
N2—C4—C5—N1	−177.6 (3)	C12—C13—C14—C15	0.0
C11—C6—C7—C8	0.0	C13—C14—C15—C10	0.0
C11—C6—C7—O1	175.81 (15)	C9—C10—C15—C14	180.0
C1—O1—C7—C6	90.0 (3)	C11—C10—C15—C14	0.0
